# Crystal structure of 2-[2-(2,5-di­chloro­benz­yloxy)-2-(furan-2-yl)eth­yl]-2*H*-indazole

**DOI:** 10.1107/S2056989016013827

**Published:** 2016-09-05

**Authors:** Özden Özel Güven, Gökhan Türk, Philip D. F. Adler, Simon J. Coles, Tuncer Hökelek

**Affiliations:** a Department of Chemistry, Bülent Ecevit University, 67100 Zonguldak, Turkey; b Department of Chemistry, Southampton University, SO17 1BJ Southampton, England; c Department of Physics, Hacettepe University, 06800 Beytepe, Ankara, Turkey

**Keywords:** crystal structure, furan, indazole, C—H⋯O inter­actions, π–π stacking

## Abstract

In the title compound, C_20_H_16_Cl_2_N_2_O_2_, the indazole ring system is approximately planar [maximum deviation = 0.033 (1) Å], its mean plane is oriented at dihedral angles of 25.04 (4) and 5.10 (4)° to the furan and benzene rings, respectively. In the crystal, pairs of C—H_ind_⋯O_bo_ (ind = indazole and bo = benz­yloxy) hydrogen bonds link the mol­ecules into centrosymmetric dimers with graph-set motif *R*_2_^2^(12). Weak C—H⋯π inter­actions is also observed. Aromatic π–π stacking between the benzene and the pyrazole rings from neighbouring mol­ecules [centroid–centroid distance = 3.8894 (7) Å] further consolidates the crystal packing.

## Abstract 2

In the title compound, the indazole ring system is oriented at dihedral angles of 25.04 (4) and 5.10 (4)° o the furan and benzene rings, respectively

## Chemical context   

Ethers such as miconazole and econazole possessing an imidazole ring have been developed for clinical uses as azole anti­fungals (Godefroi *et al.*, 1969 ▸). The crystal structures of miconazole (Peeters *et al.*, 1979 ▸) and econazole (Freer *et al.*, 1986 ▸) have previously been reported. Another azole ring system, indazole, is an important structural unit of many biologically active compounds. Some indazole derivatives have been shown to exhibit anti­fungal (Lebouvier *et al.*, 2007 ▸; Park *et al.*, 2007 ▸), anti­bacterial (Wang *et al.*, 2015 ▸), anti­proliferative (Büchel *et al.*, 2012 ▸), anti­tumor (Abbassi *et al.*, 2014 ▸) activity and act as inhibitors of nitric oxide synthase with anti­oxidant properties (Salerno *et al.*, 2012 ▸). The crystal structures of some indazole derivatives have been reported (Gerpe *et al.*, 2007 ▸; Raffa *et al.*, 2009 ▸; Boulhaoua *et al.*, 2015 ▸). In addition, the crystal structures of ketones containing an indazole group (Özel Güven *et al.*, 2013 ▸, 2014*a* ▸) and ether (Özel Güven *et al.*, 2014*b* ▸) have been described. As a continuation of our studies in this area, we synthesized the title compound and report herein its crystal structure.
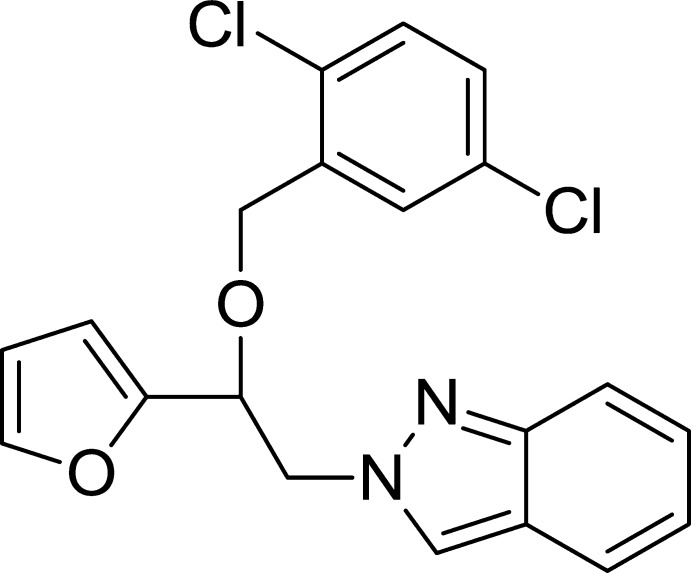


## Structural commentary   

**Figure 1:**
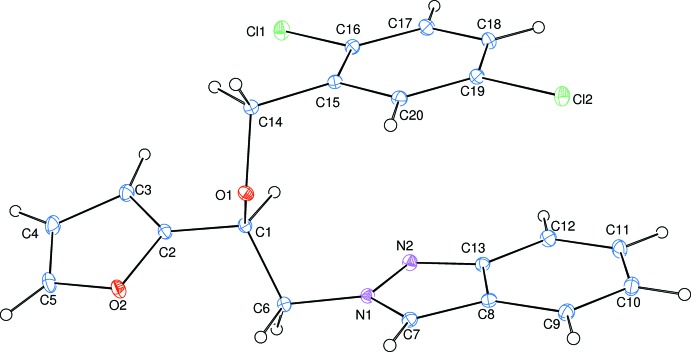
The mol­ecular structure of the title compound, showing the atom-numbering scheme. Displacement ellipsoids are drawn at the 30% probability level.

In the mol­ecule of the title compound, (Fig. 1 ▸), the bond lengths and angles are within normal ranges. The indazole (*B*; N1/N2/C7–C13) ring system is approximately planar with a maximum deviation of −0.033 (1) Å for atom C10. Its mean plane is oriented with respect to the furan (*A*; O2/C2–C5) and benzene (*C*; C15–C20) rings at dihedral angles of *A*/*B* = 25.04 (4) and *B*/*C* = 5.10 (4)°. The dihedral angle between the furan and benzene rings is 20.21 (5)°. Atom C6 is −0.054 (1) Å from the indazole ring plane, while atom C1 is 0.038 (1) Å from the furan ring plane. Atoms Cl1, Cl2 and C14 are displaced by −0.0430 (3), 0.0233 (4) and −0.016 (1) Å, respectively, to the benzene ring plane.

## Supra­molecular features   

**Figure 2:**
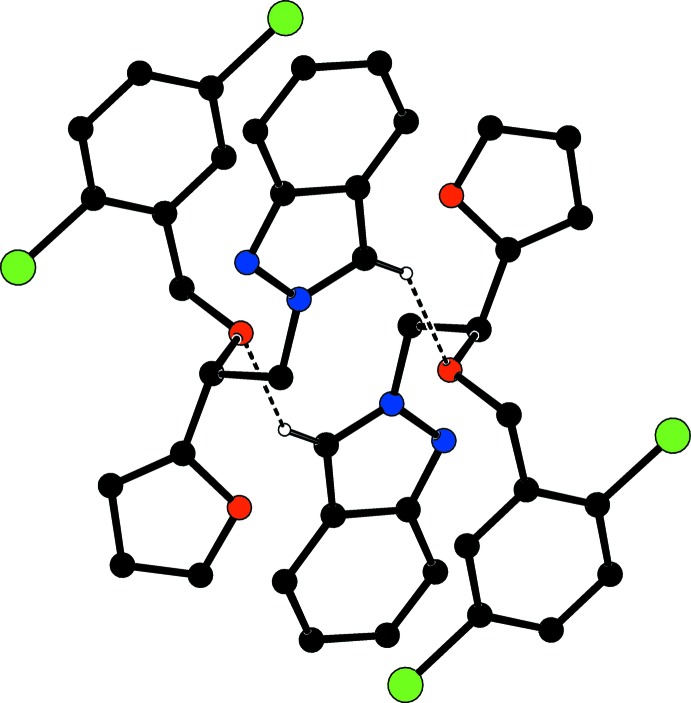
Part of the crystal structure. Inter­molecular [C—H_ind_ ⋯ O_bo_] hydrogen bonds, enclosing 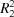
(12) ring motifs, are shown as dashed lines (see Table 1 ▸). H atoms not involved in hydrogen bonding have been omitted for clarity.

**Figure 3:**
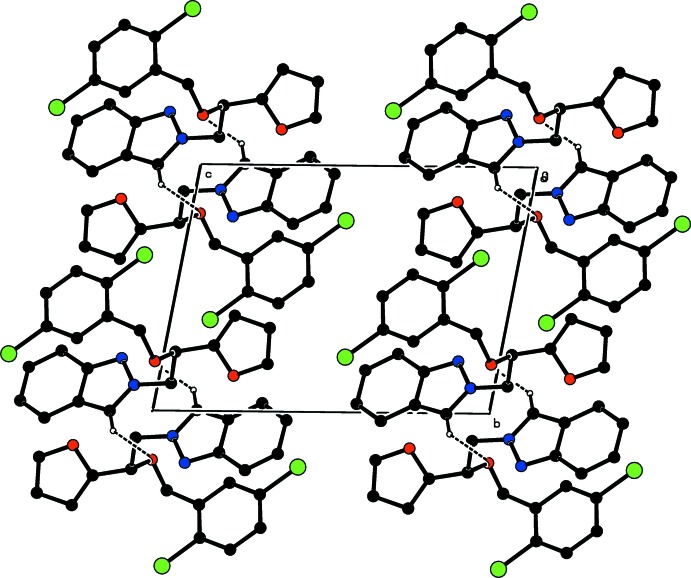
The crystal packing of the title compound, viewed down the *a* axis. Hydrogen bonds are shown as dashed lines. H atoms not involved in hydrogen bonding have been omitted for clarity.

In the crystal, pairs of C—H_ind_⋯ O_bo_ (ind = indazole and bo = benz­yloxy) hydrogen bonds (Table 1 ▸), enclosing 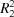
(12) ring motifs link the mol­ecules into centrosymmetric dimers (Fig. 2 ▸), which are stacked along the *a* axis and oriented along the *b*-axis direction (Fig. 3 ▸). Weak C—H⋯π inter­actions (Table 1 ▸) occur. π–π inter­actions between the pyrazole and the benzene rings, *Cg*4⋯*Cg*3^i^, of neighbouring mol­ecules further consolidate the crystal packing [centroid–centroid distance = 3.8894 (7) Å; symmetry code: (i) 2 − *x*, 2 − *y*, − *z*; *Cg*3 and *Cg*4 are the centroids of rings *C* (C15–C20) and *D* (N1/N2/C7/C8/C13)].

## Synthesis and crystallization   

The title compound was synthesized by the reaction of 1-(furan-2-yl)-2-(2*H*-indazol-2-yl)ethanol with NaH and 2,5-dichlorobenzyl bromide. NaH (16 mg, 0.394 mmol) was added in small fractions to a solution of alcohol (90 mg, 0.394 mmol) in DMF (3–4 ml). Then, 2,5-dichlorobenzyl bromide (95 mg, 0.394 mmol) was added portionwise. The mixture was stirred at room temperature for 3 h, and the excess hydride was decomposed with a small amount of methyl alcohol. After evaporation to dryness under reduced pressure, a small amount of water was added and extracted with methyl­ene chloride. The organic layer was separated, dried over anhydrous sodium sulfate, and then evaporated to dryness. The crude residue was purified by chromatography on a silica-gel column using a hexa­ne–ethyl acetate mixture (10:1) as eluent. The ether was recrystallized from 2-propanol solution to obtain colourless crystals suitable for X-ray analysis (yield; 70 mg, 46%).

## Refinement   

The experimental details including the crystal data, data collection and refinement are summarized in Table 2 ▸. The C-bound H atoms were positioned geometrically with C—H = 0.93, 0.97 and 0.98 Å, for aromatic, methyl­ene and methine H-atoms, respectively, and constrained to ride on their parent atoms, with *U*_iso_(H) = 1.2*U*_eq_(C).

## Supplementary Material

Crystal structure: contains datablock(s) I, global. DOI: 10.1107/S2056989016013827/xu5891sup1.cif

Structure factors: contains datablock(s) I. DOI: 10.1107/S2056989016013827/xu5891Isup2.hkl

Supporting information file. DOI: 10.1107/S2056989016013827/xu5891Isup3.cml

CCDC reference: 1501341

Additional supporting information: crystallographic information; 3D view; checkCIF report

## Figures and Tables

**Table 1:** Hydrogen-bond geometry (Å, °) *Cg*4 is the centroid of the N1/N2/C7/C8/C13 ring.

*D*—H⋯*A*	*D*—H	H⋯*A*	*D*⋯*A*	*D*—H⋯*A*
C7—H7⋯O1^i^	0.93	2.51	3.3062 (15)	144
C6—H6*B*⋯*Cg*4^ii^	0.97	2.84	3.4583 (13)	122

Symmetry codes: (i) 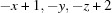; (ii) 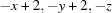.

**Table 2:** Experimental details

Crystal data	
Chemical formula	C_20_H_16_Cl_2_N_2_O_2_
*M*_r_	387.27
Crystal system, space group	Triclinic, *P* 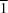
Temperature (K)	294
*a*, *b*, *c* (Å)	7.7318 (3), 9.6675 (4), 12.8299 (5)
α, β, γ (°)	76.511 (4), 76.157 (4), 73.928 (3)
*V* (Å^3^)	880.30 (6)
*Z*	2
Radiation type	Mo *K*α
μ (mm^−1^)	0.39
Crystal size (mm)	0.09 × 0.07 × 0.04

Data collection	
Diffractometer	Rigaku Saturn724+
Absorption correction	Multi-scan (*CrystalClear-SM Expert*, Rigaku, 2011 ▸)
*T*_min_, *T*_max_	0.968, 0.985
No. of measured, independent and observed [*I* > 2σ(*I*)] reflections	8400, 4278, 3813
*R*_int_	0.025
(sin θ/λ)_max_ (Å^−1^)	0.674

Refinement	
*R*[*F* ^2^ > 2σ(*F* ^2^)], *wR*(*F* ^2^), *S*	0.031, 0.083, 1.05
No. of reflections	4278
No. of parameters	235
H-atom treatment	H-atom parameters constrained
Δρ_max_, Δρ_min_ (e Å^−3^)	0.35, −0.23

Computer programs: *CrystalClear-SM Expert* (Rigaku, 2011 ▸), *SHELXS97* and *SHELXL97* (Sheldrick, 2008 ▸), *ORTEP-3 for Windows* and *WinGX* (Farrugia, 2012 ▸) and *PLATON* (Spek, 2009 ▸).

## supplementary crystallographic information

### Crystal data

**Table:**

C_20_H_16_Cl_2_N_2_O_2_	*Z* = 2
*M**_r_* = 387.27	*F*(000) = 400
Triclinic, *P*1	*D*_x_ = 1.461 Mg m^−^^3^
Hall symbol: -P 1	Mo *K*α radiation, λ = 0.71073 Å
*a* = 7.7318 (3) Å	Cell parameters from 7516 reflections
*b* = 9.6675 (4) Å	θ = 3.0–28.6°
*c* = 12.8299 (5) Å	µ = 0.39 mm^−^^1^
α = 76.511 (4)°	*T* = 294 K
β = 76.157 (4)°	Block, colorless
γ = 73.928 (3)°	0.09 × 0.07 × 0.04 mm
*V* = 880.30 (6) Å^3^	

### Data collection

**Table:**

Rigaku Saturn724+ diffractometer	3813 reflections with *I* > 2σ(*I*)
Radiation source: fine-focus sealed tube	*R*_int_ = 0.025
Graphite monochromator	θ_max_ = 28.6°, θ_min_ = 3.0°
ω scans	*h* = −6→10
Absorption correction: multi-scan (*CrystalClear-SM Expert*, Rigaku, 2011)	*k* = −13→13
*T*_min_ = 0.968, *T*_max_ = 0.985	*l* = −17→17
8400 measured reflections	3 standard reflections every 120 min
4278 independent reflections	intensity decay: 1%

### Refinement

**Table:**

Refinement on *F*^2^	Primary atom site location: structure-invariant direct methods
Least-squares matrix: full	Secondary atom site location: difference Fourier map
*R*[*F*^2^ > 2σ(*F*^2^)] = 0.031	Hydrogen site location: inferred from neighbouring sites
*wR*(*F*^2^) = 0.083	H-atom parameters constrained
*S* = 1.05	*w* = 1/[σ^2^(*F*_o_^2^) + (0.0387*P*)^2^ + 0.3381*P*] where *P* = (*F*_o_^2^ + 2*F*_c_^2^)/3
4278 reflections	(Δ/σ)_max_ = 0.001
235 parameters	Δρ_max_ = 0.35 e Å^−^^3^
0 restraints	Δρ_min_ = −0.23 e Å^−^^3^

### Special details

**Table:**

Geometry. All esds (except the esd in the dihedral angle between two l.s. planes) are estimated using the full covariance matrix. The cell esds are taken into account individually in the estimation of esds in distances, angles and torsion angles; correlations between esds in cell parameters are only used when they are defined by crystal symmetry. An approximate (isotropic) treatment of cell esds is used for estimating esds involving l.s. planes.
Refinement. Refinement of F^2^ against ALL reflections. The weighted R-factor wR and goodness of fit S are based on F^2^, conventional R-factors R are based on F, with F set to zero for negative F^2^. The threshold expression of F^2^ > 2sigma(F^2^) is used only for calculating R-factors(gt) etc. and is not relevant to the choice of reflections for refinement. R-factors based on F^2^ are statistically about twice as large as those based on F, and R- factors based on ALL data will be even larger.

### Fractional atomic coordinates and isotropic or equivalent isotropic displacement parameters (Å^2^)

**Table:**

	*x*	*y*	*z*	*U*_iso_*/*U*_eq_	
Cl1	0.24682 (4)	0.62858 (3)	0.88313 (3)	0.02337 (9)	
Cl2	0.49082 (5)	0.22144 (3)	0.53654 (2)	0.02445 (9)	
O1	0.44963 (11)	0.19772 (8)	0.97438 (7)	0.01525 (17)	
O2	0.31136 (12)	0.14245 (9)	1.21688 (7)	0.02059 (19)	
N1	0.15767 (13)	0.10240 (10)	0.92745 (8)	0.01489 (19)	
N2	0.03507 (13)	0.21036 (11)	0.87622 (8)	0.0175 (2)	
C1	0.26260 (15)	0.23752 (12)	1.03067 (9)	0.0143 (2)	
H1	0.1970	0.3262	0.9884	0.017*	
C2	0.25595 (15)	0.26448 (13)	1.14153 (9)	0.0161 (2)	
C3	0.21044 (18)	0.38619 (14)	1.18630 (10)	0.0211 (2)	
H3	0.1687	0.4820	1.1524	0.025*	
C4	0.23944 (19)	0.33774 (15)	1.29692 (11)	0.0254 (3)	
H4	0.2204	0.3962	1.3487	0.031*	
C5	0.29944 (18)	0.19136 (15)	1.31093 (10)	0.0236 (3)	
H5	0.3288	0.1316	1.3755	0.028*	
C6	0.17821 (16)	0.11038 (12)	1.03596 (9)	0.0160 (2)	
H6A	0.2559	0.0195	1.0659	0.019*	
H6B	0.0592	0.1233	1.0839	0.019*	
C7	0.25036 (15)	−0.00406 (12)	0.87016 (10)	0.0156 (2)	
H7	0.3402	−0.0856	0.8913	0.019*	
C8	0.18475 (15)	0.03232 (12)	0.77239 (10)	0.0156 (2)	
C9	0.21674 (17)	−0.03471 (13)	0.68025 (10)	0.0188 (2)	
H9	0.3022	−0.1232	0.6750	0.023*	
C10	0.11867 (17)	0.03397 (14)	0.59908 (10)	0.0214 (2)	
H10	0.1362	−0.0095	0.5387	0.026*	
C11	−0.00995 (17)	0.17116 (14)	0.60570 (10)	0.0222 (3)	
H11	−0.0724	0.2161	0.5485	0.027*	
C12	−0.04425 (17)	0.23857 (13)	0.69382 (10)	0.0203 (2)	
H12	−0.1283	0.3281	0.6970	0.024*	
C13	0.05251 (15)	0.16753 (12)	0.78018 (9)	0.0158 (2)	
C14	0.51911 (16)	0.32122 (12)	0.91196 (9)	0.0157 (2)	
H14A	0.6518	0.2928	0.8940	0.019*	
H14B	0.4856	0.3981	0.9550	0.019*	
C15	0.44190 (15)	0.37805 (12)	0.80800 (9)	0.0149 (2)	
C16	0.32003 (16)	0.51321 (12)	0.78692 (10)	0.0169 (2)	
C17	0.24998 (17)	0.55987 (13)	0.69043 (10)	0.0204 (2)	
H17	0.1687	0.6505	0.6783	0.024*	
C18	0.30253 (17)	0.47018 (14)	0.61306 (10)	0.0210 (2)	
H18	0.2581	0.5002	0.5482	0.025*	
C19	0.42271 (16)	0.33464 (13)	0.63373 (10)	0.0180 (2)	
C20	0.49107 (16)	0.28785 (13)	0.72976 (9)	0.0166 (2)	
H20	0.5699	0.1961	0.7422	0.020*	

### Atomic displacement parameters (Å^2^)

**Table:**

	*U*^11^	*U*^22^	*U*^33^	*U*^12^	*U*^13^	*U*^23^
Cl1	0.02851 (16)	0.01783 (14)	0.02403 (16)	0.00089 (11)	−0.00838 (12)	−0.00825 (11)
Cl2	0.03293 (17)	0.02802 (16)	0.01613 (15)	−0.01132 (13)	−0.00224 (12)	−0.00855 (11)
O1	0.0145 (4)	0.0151 (4)	0.0142 (4)	−0.0024 (3)	−0.0014 (3)	−0.0015 (3)
O2	0.0261 (4)	0.0211 (4)	0.0132 (4)	−0.0024 (3)	−0.0051 (3)	−0.0027 (3)
N1	0.0148 (4)	0.0151 (4)	0.0151 (5)	−0.0031 (3)	−0.0037 (4)	−0.0026 (3)
N2	0.0166 (5)	0.0168 (5)	0.0189 (5)	−0.0014 (4)	−0.0057 (4)	−0.0032 (4)
C1	0.0144 (5)	0.0148 (5)	0.0129 (5)	−0.0022 (4)	−0.0021 (4)	−0.0026 (4)
C2	0.0151 (5)	0.0191 (5)	0.0135 (5)	−0.0041 (4)	−0.0026 (4)	−0.0016 (4)
C3	0.0253 (6)	0.0211 (6)	0.0184 (6)	−0.0050 (5)	−0.0049 (5)	−0.0060 (4)
C4	0.0312 (7)	0.0310 (7)	0.0177 (6)	−0.0082 (5)	−0.0044 (5)	−0.0105 (5)
C5	0.0263 (6)	0.0324 (7)	0.0125 (5)	−0.0060 (5)	−0.0049 (5)	−0.0045 (5)
C6	0.0182 (5)	0.0168 (5)	0.0134 (5)	−0.0049 (4)	−0.0028 (4)	−0.0027 (4)
C7	0.0152 (5)	0.0146 (5)	0.0172 (5)	−0.0032 (4)	−0.0032 (4)	−0.0033 (4)
C8	0.0143 (5)	0.0159 (5)	0.0169 (5)	−0.0053 (4)	−0.0020 (4)	−0.0023 (4)
C9	0.0196 (5)	0.0195 (5)	0.0187 (6)	−0.0059 (4)	−0.0021 (4)	−0.0058 (4)
C10	0.0222 (6)	0.0268 (6)	0.0183 (6)	−0.0083 (5)	−0.0034 (5)	−0.0071 (5)
C11	0.0225 (6)	0.0273 (6)	0.0187 (6)	−0.0061 (5)	−0.0093 (5)	−0.0018 (5)
C12	0.0186 (5)	0.0201 (6)	0.0219 (6)	−0.0027 (4)	−0.0067 (5)	−0.0026 (5)
C13	0.0150 (5)	0.0168 (5)	0.0163 (5)	−0.0050 (4)	−0.0025 (4)	−0.0030 (4)
C14	0.0158 (5)	0.0163 (5)	0.0154 (5)	−0.0047 (4)	−0.0030 (4)	−0.0024 (4)
C15	0.0143 (5)	0.0164 (5)	0.0148 (5)	−0.0058 (4)	−0.0021 (4)	−0.0022 (4)
C16	0.0186 (5)	0.0158 (5)	0.0167 (5)	−0.0046 (4)	−0.0032 (4)	−0.0034 (4)
C17	0.0225 (6)	0.0180 (5)	0.0201 (6)	−0.0039 (4)	−0.0075 (5)	0.0000 (4)
C18	0.0251 (6)	0.0246 (6)	0.0149 (5)	−0.0093 (5)	−0.0069 (5)	0.0009 (4)
C19	0.0201 (5)	0.0216 (6)	0.0140 (5)	−0.0094 (5)	−0.0004 (4)	−0.0042 (4)
C20	0.0169 (5)	0.0169 (5)	0.0161 (5)	−0.0053 (4)	−0.0020 (4)	−0.0026 (4)

### Geometric parameters (Å, º)

**Table:**

Cl1—C16	1.7453 (12)	C8—C13	1.4269 (16)
Cl2—C19	1.7416 (12)	C9—C8	1.4169 (17)
O1—C1	1.4431 (13)	C9—C10	1.3699 (17)
O1—C14	1.4348 (13)	C9—H9	0.9300
O2—C2	1.3770 (14)	C10—C11	1.4272 (18)
O2—C5	1.3701 (15)	C10—H10	0.9300
N1—C6	1.4601 (14)	C11—H11	0.9300
N1—C7	1.3437 (15)	C12—C11	1.3703 (18)
N2—N1	1.3603 (13)	C12—C13	1.4199 (16)
N2—C13	1.3548 (15)	C12—H12	0.9300
C1—C6	1.5248 (15)	C14—H14A	0.9700
C1—H1	0.9800	C14—H14B	0.9700
C2—C1	1.4913 (16)	C15—C14	1.5149 (15)
C2—C3	1.3519 (17)	C15—C16	1.3942 (16)
C3—C4	1.4380 (17)	C15—C20	1.3981 (16)
C3—H3	0.9300	C16—C17	1.3980 (16)
C4—H4	0.9300	C17—H17	0.9300
C5—C4	1.3445 (19)	C18—C17	1.3841 (18)
C5—H5	0.9300	C18—C19	1.3901 (18)
C6—H6A	0.9700	C18—H18	0.9300
C6—H6B	0.9700	C19—C20	1.3861 (16)
C7—H7	0.9300	C20—H20	0.9300
C8—C7	1.3980 (16)		
			
C14—O1—C1	113.25 (8)	C10—C9—H9	120.8
C5—O2—C2	106.39 (10)	C9—C10—C11	121.05 (12)
N2—N1—C6	119.34 (9)	C9—C10—H10	119.5
C7—N1—N2	114.58 (10)	C11—C10—H10	119.5
C7—N1—C6	126.08 (10)	C10—C11—H11	119.0
C13—N2—N1	103.16 (9)	C12—C11—C10	121.96 (11)
O1—C1—C2	110.75 (9)	C12—C11—H11	119.0
O1—C1—C6	105.96 (9)	C11—C12—C13	117.94 (11)
O1—C1—H1	109.4	C11—C12—H12	121.0
C2—C1—C6	111.85 (9)	C13—C12—H12	121.0
C2—C1—H1	109.4	N2—C13—C8	111.76 (10)
C6—C1—H1	109.4	N2—C13—C12	128.06 (11)
O2—C2—C1	115.82 (10)	C12—C13—C8	120.17 (11)
C3—C2—O2	110.25 (10)	O1—C14—C15	110.37 (9)
C3—C2—C1	133.91 (11)	O1—C14—H14A	109.6
C2—C3—C4	106.19 (11)	O1—C14—H14B	109.6
C2—C3—H3	126.9	C15—C14—H14A	109.6
C4—C3—H3	126.9	C15—C14—H14B	109.6
C3—C4—H4	126.7	H14A—C14—H14B	108.1
C5—C4—C3	106.63 (11)	C16—C15—C14	123.97 (10)
C5—C4—H4	126.7	C16—C15—C20	117.85 (11)
O2—C5—H5	124.7	C20—C15—C14	118.16 (10)
C4—C5—O2	110.55 (11)	C15—C16—Cl1	120.24 (9)
C4—C5—H5	124.7	C15—C16—C17	121.87 (11)
N1—C6—C1	110.63 (9)	C17—C16—Cl1	117.87 (9)
N1—C6—H6A	109.5	C16—C17—H17	120.3
N1—C6—H6B	109.5	C18—C17—C16	119.50 (11)
C1—C6—H6A	109.5	C18—C17—H17	120.3
C1—C6—H6B	109.5	C17—C18—C19	119.07 (11)
H6A—C6—H6B	108.1	C17—C18—H18	120.5
N1—C7—C8	106.25 (10)	C19—C18—H18	120.5
N1—C7—H7	126.9	C18—C19—Cl2	119.43 (9)
C8—C7—H7	126.9	C20—C19—Cl2	119.11 (9)
C7—C8—C9	135.24 (11)	C20—C19—C18	121.45 (11)
C7—C8—C13	104.23 (10)	C15—C20—H20	119.9
C9—C8—C13	120.48 (11)	C19—C20—C15	120.25 (11)
C8—C9—H9	120.8	C19—C20—H20	119.9
C10—C9—C8	118.36 (11)		
			
C14—O1—C1—C2	−89.83 (11)	C7—C8—C13—N2	−1.03 (13)
C14—O1—C1—C6	148.69 (9)	C7—C8—C13—C12	−179.97 (10)
C1—O1—C14—C15	−75.87 (11)	C9—C8—C13—N2	176.94 (10)
C5—O2—C2—C1	178.44 (10)	C9—C8—C13—C12	−2.01 (17)
C5—O2—C2—C3	−0.06 (13)	C10—C9—C8—C7	177.63 (13)
C2—O2—C5—C4	−0.05 (14)	C10—C9—C8—C13	0.43 (17)
N2—N1—C6—C1	−67.74 (13)	C8—C9—C10—C11	1.28 (18)
C7—N1—C6—C1	112.18 (12)	C9—C10—C11—C12	−1.5 (2)
N2—N1—C7—C8	−0.85 (13)	C13—C12—C11—C10	−0.12 (19)
C6—N1—C7—C8	179.22 (10)	C11—C12—C13—N2	−176.94 (12)
C13—N2—N1—C6	−179.86 (10)	C11—C12—C13—C8	1.81 (17)
C13—N2—N1—C7	0.21 (13)	C16—C15—C14—O1	112.32 (12)
N1—N2—C13—C8	0.53 (12)	C20—C15—C14—O1	−65.60 (13)
N1—N2—C13—C12	179.37 (11)	C14—C15—C16—Cl1	−0.09 (16)
O1—C1—C6—N1	−69.01 (11)	C14—C15—C16—C17	−178.88 (11)
C2—C1—C6—N1	170.22 (9)	C20—C15—C16—Cl1	177.83 (9)
O2—C2—C1—O1	−71.29 (12)	C20—C15—C16—C17	−0.95 (17)
O2—C2—C1—C6	46.66 (13)	C14—C15—C20—C19	179.49 (10)
C3—C2—C1—O1	106.75 (15)	C16—C15—C20—C19	1.44 (17)
C3—C2—C1—C6	−135.30 (14)	Cl1—C16—C17—C18	−178.91 (9)
O2—C2—C3—C4	0.13 (14)	C15—C16—C17—C18	−0.10 (19)
C1—C2—C3—C4	−177.99 (13)	C19—C18—C17—C16	0.66 (18)
C2—C3—C4—C5	−0.16 (15)	C17—C18—C19—Cl2	−179.60 (9)
O2—C5—C4—C3	0.13 (16)	C17—C18—C19—C20	−0.16 (18)
C9—C8—C7—N1	−176.43 (13)	Cl2—C19—C20—C15	178.52 (9)
C13—C8—C7—N1	1.08 (12)	C18—C19—C20—C15	−0.92 (18)

### Hydrogen-bond geometry (Å, º)

Cg4 is the centroid of the N1/N2/C7/C8/C13 ring.

**Table:**

*D*—H···*A*	*D*—H	H···*A*	*D*···*A*	*D*—H···*A*
C7—H7···O1^i^	0.93	2.51	3.3062 (15)	144
C6—H6*B*···*Cg*4^ii^	0.97	2.84	3.4583 (13)	122

Symmetry codes: (i) −*x*+1, −*y*, −*z*+2; (ii) −*x*+2, −*y*+2, −*z*.
